# Tumor budding predicts lymph node metastasis in squamous cell carcinoma of the lip

**DOI:** 10.1186/s13005-025-00553-2

**Published:** 2025-11-03

**Authors:** S. Leypold, J. Riese, C. Cacchi, K. Wermker, O. Vladu, D. Jonigk, F. Hölzle, M. Klein

**Affiliations:** 1 Institute of Pathology, School of Medicine, University Hospital RWTH Aachen, Pauwelsstrasse 30, Aachen, 52074 Germany; 2 Department of Oral and Cranio-Maxillofacial Surgery, Klinikum Osnabrück GmbH, Am Finkenhügel 1, Osnabrück, 49076 Germany; 3 Department of Oral & Maxillofacial Surgery, School of Medicine, University Hospital RWTH Aachen, Pauwelsstrasse 30, Aachen, 52074 Germany; 4 German Center for Lung Research (DZL), BREATH Hanover, Hannover, 30625 Germany

**Keywords:** Tumor budding, Lip cancer, LSCC, Cancer, Tumor marker, CSCC, HNSCC

## Abstract

**Background:**

Squamous cell carcinoma of the lip (LSCC) is a relatively rare malignancy. The criteria for performing a neck dissection remain unclear, as reliable predictors for lymph node metastasis (LNM) have not been fully established. This study aimed to identify risk factors for LNM to guide the indication for elective neck dissection.

**Methods:**

A total of 57 patients with LSCC were evaluated based on 81 clinical and pathological parameters, including three previously published pathological grading systems. Statistical analyses focused on identifying the most relevant and independent predictors of LNM using univariate and multivariate logistic regression, supplemented by the LASSO algorithm for feature selection.

**Results:**

Tumor size (OR 1.008, *p* = 0.85) and peritumoral tumor budding (OR 1.43, *p* = 0.059) emerged as the most relevant independent predictors of LNM. Notably, the number of peritumoral tumor buds was significantly higher in lymph node-positive (pN +) patients compared to lymph node-negative (pN −) patients (*p <* 0.01). Receiver operating characteristic (ROC) curve analysis demonstrated that peritumoral tumor budding outperformed other classification systems, with the highest area under the curve (AUC = 0.86).

**Conclusion:**

Peritumoral tumor budding shows strong predictive potential for lymph node metastasis in LSCC, offering valuable insights for the preoperative evaluation and indication for elective neck dissection.

**Supplementary Information:**

The online version contains supplementary material available at 10.1186/s13005-025-00553-2.

## Introduction

The lymph node metastasis (LNM) rate for cutaneous squamous cell carcinoma (cSCC) is relatively low, reported in the literature at 3.7% [1]. Consequently, the benefit of elective neck dissection in cSCC is deemed minimal and is generally not recommended [2]. In contrast, more aggressive cSCC localizations such as the lip (LSCC) and the ear show higher metastasis and recurrence rates (8–25%) compared to other localizations of the skin [3]. LNM is associated with significantly poorer outcomes, including reduced 5-year survival rates for patients [4, 5].

In cases of clinically suspected lymph node metastasis (cN +), elective (END) or therapeutic neck dissection (TND) is typically performed. However, determining the indication for neck dissection in clinically unremarkable regional lymph nodes (cN0) remains challenging, particularly for tumors such as LSCC. LSCC exhibits a low rate of occult metastasis compared to squamous cell carcinoma of the head and neck region (HNSCC) but a significantly higher rate compared to cSCC.

For HNSCC, a threshold of 20% occult metastasis is generally considered necessary for the benefits of END to outweigh the risks.^6^ Based on reported metastasis rates, LSCC may or may not meet this threshold, leading to frequent discussions in interdisciplinary tumor conferences about the potential for over- or undertreatment with END. It is widely recognized that neck dissection can significantly impact quality of life [6, 7].

For LSCC, immunohistochemical or morphological tumor markers identified in a biopsy could assist in determining the indication for elective neck dissection in clinically unremarkable (cN0) necks [8–10]. Risk assessment for LNM has predominantly focused on pathological grading systems. Wermker et al. demonstrated that grading can serve as a predictive factor for LNM [11]. However, various grading systems exist for LSCC, and to date, no definitive evidence suggests that any single system offers a clear advantage over the others [12].

The aim of this study was to identify potential predictors of LNM in LSCC and evaluate their ability to distinguish pN + from pN0 in clinically unremarkable (cN0) necks. A secondary objective was to compare the predictive value of various grading systems for LNM.

## Material and methods

### Patients and clinical data

This study included only patients with histologically confirmed LSCC aged over 18 years. Patients with tumor recurrence or secondary cSCC were also included. Patients with incomplete data sets were excluded. All participants underwent preoperative staging. Tumor characteristics, follow-up data, and survival-related information were retrospectively collected from the hospital’s database system. The following clinical parameters were recorded: initial diagnosis date, American Society of Anesthesiologists (ASA) score, immunosuppression status, tumor location (upper or lower lip), primary recurrence, secondary SCC, time from initial diagnosis to follow-up, and progression-relevant factors, including local recurrence, LNM during follow-up, distant metastasis (DM) during follow-up, and cause of death (disease-specific or other causes).

### Histopathologicals specimens

Formalin-fixed paraffin-embedded (FFPE) samples of *n =* 57 cases of LSCC were retrieved from the archive of the Department of Pathology at RWTH Aachen University, Germany. Only cases derived from entire surgical resections, with paraffine blocks containing sufficient material for histopathological analysis, were included in this study. Three-micrometer thick sections were obtained from the FFPE tissue blocks and stained with hematoxylin & eosin (H&E) using an automated stainer system (Tissue-Tek-Prisma®, Sakura, Umkirch, Germany). An experienced pathologist (SL), who was unaware of the clinical data and study outcomes, analyzed the specimens using a light microscope (Zeiss Axio Lab.A1 microscope, Oberkochen, Germany).

### Histomorphological parameters

In total 30 histomorphological parameters were analyzed. These parameters included: histological tumor localization, tumor size (mm), depth of invasion, measured from the stratum granulosum and the stratum basale (mm), pathological T-stage (pT) according to the current 8th TNM UICC classification for oral cavity and skin, tumor thickness (mm), presence of ulceration, necrosis, muscle infiltration, salivary gland infiltration and bone infiltration, tumor-stroma ratio (%), presence of lymph vessel invasion (L), venous invasion (V) and intra- and peritumoral perineural invasion (Pn), mitosis per 2 mm^2^, eosinophilia per 2 mm^2^, presence of solar elastosis, presence of a satellite tumor nodule at least 1 mm away from the main tumor [13]. Resection status (R) and the minimum distance to the resection margin (mm) were also assessed.

**Fig. 1:**
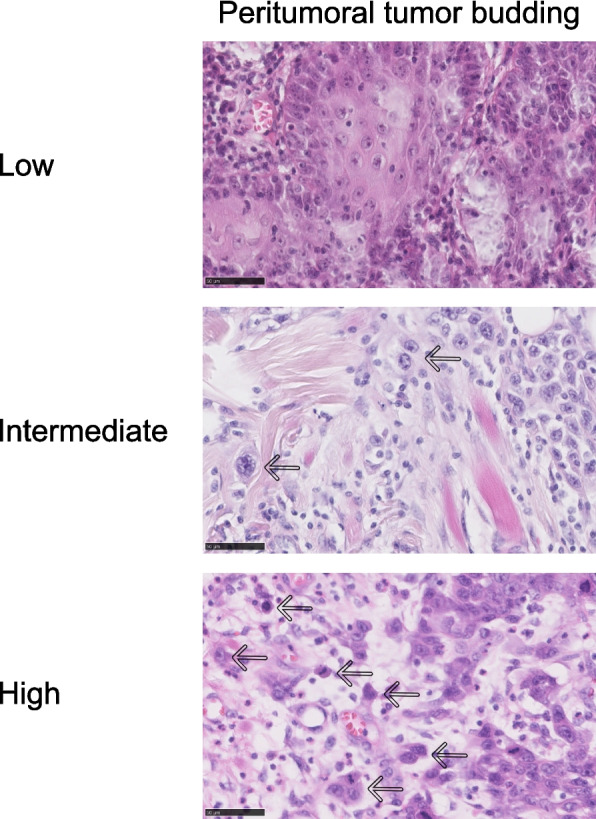
Representative histological images. The different TB scores in histological HE sections of LSCC are shown: low TB (0–4 buds), intermediate TB (5–9 buds) and high TB (> 10 buds). HE; magnification 40x; arrows mark tumor buds (buds = 1—max. 4 cells). A scale bar of 50 µm is shown in each image

Furthermore, intra- (ITB) and peritumoral tumor budding (PTB) were evaluated. Recently, PTB—defined as the presence of isolated tumor cells or small cell clusters (< 5 cells) detached from the tumor invasion front (TIF) [14] has gained prominence as a predictor across different cancer entities, including HNSCC [10, 15]. For this analysis, 10 fields were examined at 10 × magnification to identify the local hotspot area. This area was then inspected at 20 × magnification, and tumor buds – defined as a single tumor cell or a cluster of up to four tumor cells – were quantified [16]. Additionally, TB was categorized as follows: low TB (0–4 buds), intermediate TB (5–9 buds) and high TB ≥ 10 buds); (Fig. 1) [10, 17]. All TB assessments were performed by a single experienced pathologist (SL), blinded to all clinical and outcome data, following a standardized protocol adapted from the “International Tumor Budding Consensus Conference (ITBCC) 2016 recommendations” [16]. In cases where the invasion front of the tumor was obscured by inflammatory cells, immunohistochemistry-using pan-cytokeratin (Cytokeratin, clones AE1/AE3, DAKO, Santa Clara, CA, United States) was performed to identify the hotspot area. Additionally, three established grading systems for HNSCCs were used: Boxberg grading, Agaimy grading and Bryne grading [18–20]. The parameters defining the grading systems are summarized in Supplementary Table 1.

To assess the pathological lymph node status (pN), the size of metastases and the presence of extranodal extension were evaluated. Finally, the lymph node ratio (LNR) was calculated as the ratio of metastatic lymph nodes to all lymph nodes in the neck dissection, and classification for all specimens was performed according to the current 8th TNM UICC classification separately for oral cavity and skin.

### Statistical analysis

For group comparisons involving non-parametric data, we used the chi-squared test. For parametric data, normality was first assessed using the Shapiro–Wilk test. In all comparisons presented in this study, the data were not normally distributed; therefore, the Mann–Whitney U test was applied.

In the regression models, covariates were initially selected by performing univariate logistic regression for each parameter. Parameters with a p-value below 0.1 in the Wald test were retained for further analysis [21]. Next, parameters that contained inherent information on lymph node metastasis (e.g. metastasis size) were excluded. The remaining parameters were then included in a LASSO (least absolute shrinkage and selection operator) regression with standard settings (alpha = 1.0, maximum iterations = 1000, optimization tolerance = 10⁻^1^, and a cyclic selection algorithm). Subsequently, a multivariate logistic regression model was built using the parameters identified in the LASSO analysis, specifically tumor size (in mm) and PTB (count). We calculated the probability of lymph node metastasis using the LogisticRegression module from scikit-learn for various univariate models. ROC curves with corresponding AUC values were also generated using specific scikit-learn packages. All statistical analyses were conducted in Python (version 3.11.7) within a local JupyterLab environment (version 4.0.11). Details on the specific package versions used in this study are provided in Supplementary Table 2. Figures were created in Inkscape (version 1.1).

## Results

### Patient characteristics and feature selection

**Fig. 2:**
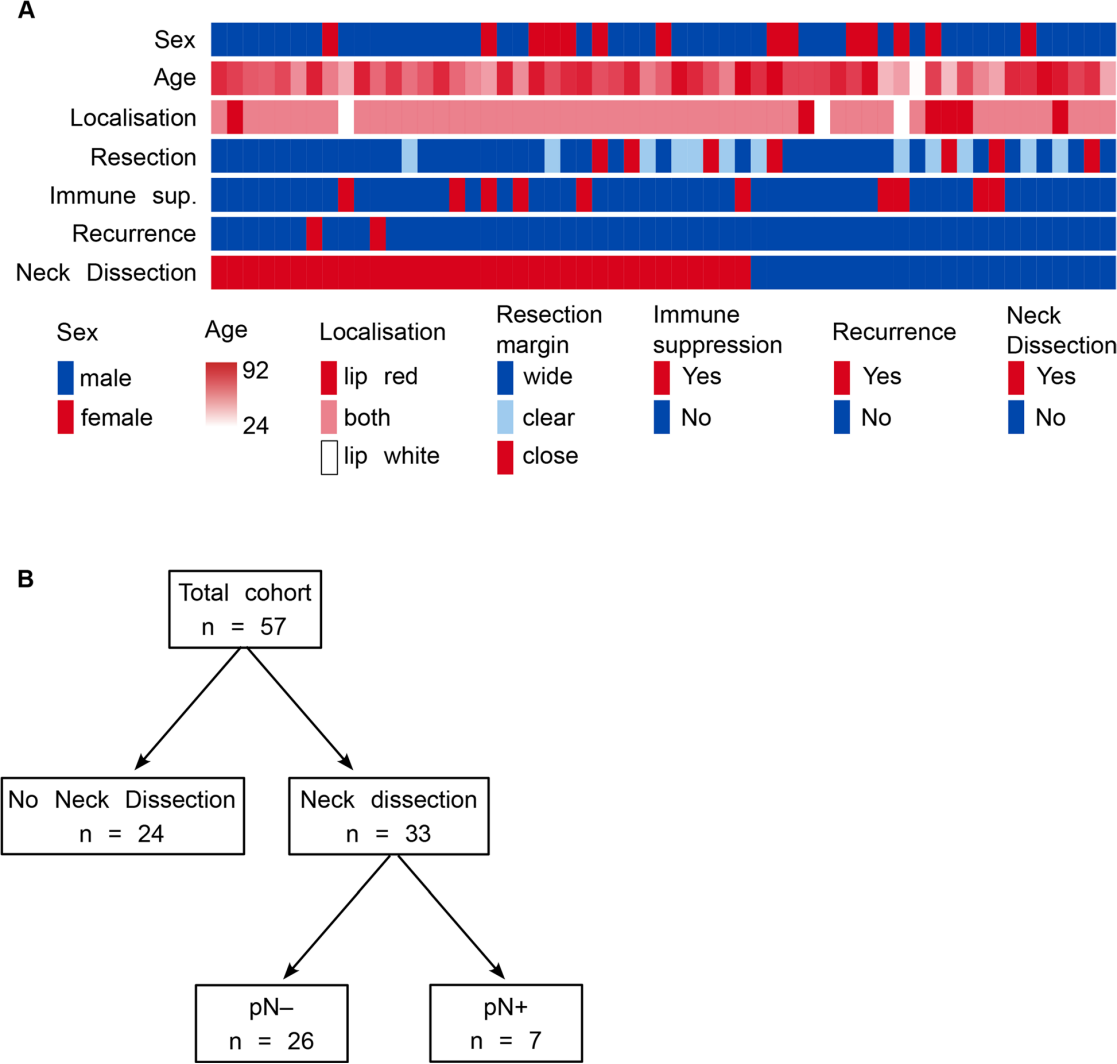
**a** Heatmap of patient characteristics grouped by neck dissection status. Age was normalized using min–max scaling across the entire cohort. Resection margins were defined as “close” (< 3 mm), “clear” (3 to 5 mm), and “wide” (> 5 mm).** b** Schematic overview of the total patient cohort with LSCC, categorized into patients who underwent END and those who did not. Patients who underwent END were further stratified into those with lymph node metastasis (pN^+^) and those without lymph node metastasis (pN.^–^)

**Fig. 3:**
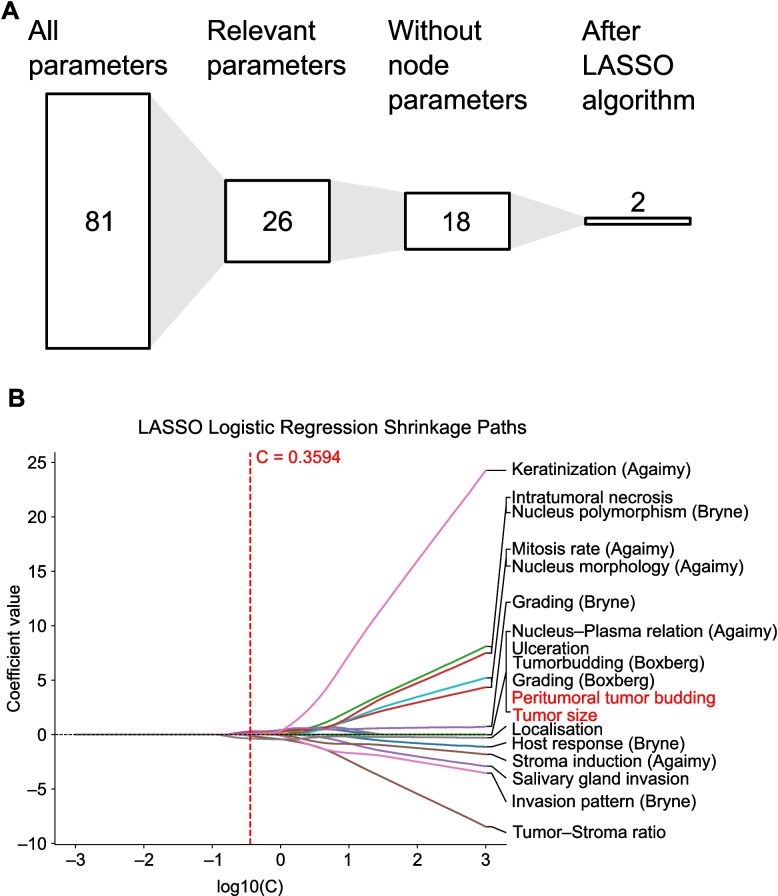
**a** Feature selection workflow for identifying parameters relevant to predicting LNM. Initially, 81 parameters were considered, with 26 relevant parameters identified using the Mann–Whitney U test and Chi-square test. Eight parameters directly associated with LNM (e.g., number of positive lymph nodes and presence of extranodal growth) were excluded from further analysis. **b** LASSO paths of 18 predictors with coefficient estimates plotted against log10(C) with the optimal regularization parameter (C = 0.3594) is indicated, with significant predictors highlighted in red

A total of 57 patients were included in the study (see overview of the study population in Tables 1 and 2, Supplementary Table 3 and Fig. 2 A). Of these, 33 underwent END, with 7 patients showing a pN + status and 26 patients classified as pN − (Fig. 2B). Univariate analysis identified 26 relevant parameters out of 81 (Fig. 3A). After excluding parameters directly associated with lymph node metastasis (LNM), such as metastasis size, 18 parameters were input into the LASSO algorithm. This analysis identified two independent parameters for assessing lymph node status: tumor size and PTB (Fig. 3B).

**Table 1:** Clinical patient data

	**Total cohort *n =* 57**	**Neck Dissection *n =* 33**	**No N.D. *n =* 24**
Age* [years]	75 ± 23	72 ± 20	76 ± 27
Gender^†^			
• Male	43	26	17
• Female	14	7	7
Localization^†^			
• Lip white	3	1	2
• Lip red	6	1	5
• Combination	47	31	16
Lip			
• Upper	2	0	2
• Lower	54	32	22
• Both	1	1	0
Recurrence			
• Local	2	1	1
• Lymph node	2	2	0
• None	53	31	23
Follow-up [months]*	12.0 ± 28.5	11.0 ± 29.5	13.0 ± 25.25

**Table 2:** Pathological patient data

	**Total cohort *n =* 57**	**Neck Dissection *n =* 33**	**No N.D *n =* 24**
Tumor size [mm]^*^	15 ± 12	18 ± 12	11.5 ± 10.0
Tumor invasion depth [mm]^*^			
• from Str. granulosum	3.0 ± 4.5	4.0 ± 5.5	2.0 ± 1.75
• from Str. basale	3.0 ± 4.75	4.0 ± 5.0	2.0 ± 2.0
T stage (skin)^†^			
• pT1	34	16	18
• pT2	9	5	4
• pT3	14	12	2
T stage (oral cavity)^†^			
• pT1	35	17	18
• pT2	16	11	5
• pT3	6	5	1
Clinical N stage^†^			
• pN0	47	23	24
• pN1	4	4	0
• pN2	3	3	0
• pN3	0	0	0
Pathological N stage^†^			
• pN0	26	26	–
• pN1	4	4	–
• pN2	2	2	–
• pN3	1	1	–
Lymph angioinvasion^†^			
• L0	56	33	23
• L1	1	0	1
Venous angioinvasion^†^			
• V0	56	33	23
• V1	1	0	1
Perineural growth (outside tumor)^†^			
• Pn0	51	29	22
• Pn1	6	4	2

^*^Median ± interquartile range (IQR)

^†^Count data

### PTB is the strongest, independent predictor of lymph node metastasis

**Fig. 4:**
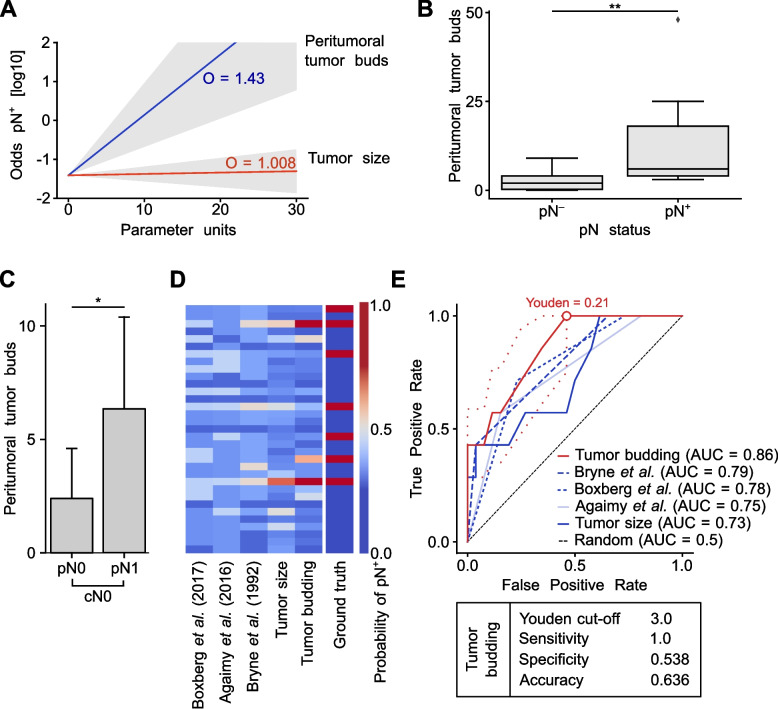
**a** Visualization of the odds ratio (OR) of LNM (pN +) of tumor size (red) and PTB (blue). **b** Boxplot showing the number of peritumoral tumor buds in patients who underwent END without LNM (pN–) versus those with LNM (pN +). Statistical comparison was performed using the Mann–Whitney U test, with ** *p <* 0.01. **c** Boxplot showing the number of peritumoral tumor buds in patients with cN0 neck who underwent END without LNM (pN–) compared to patients with LNM (pN +). The statistical comparison was performed using the Mann–Whitney U test, with * *p <* 0.05. **d** Heatmap illustrating predicted lymph node status based on univariate logistic regression models. Each row corresponds to a patient, with cell color indicating the probability of LNM (blue = low probability, red = high probability). “Ground truth” denotes actual END results (blue = pN0, red = pN +). Only patients who underwent END are included. **e** ROC (Receiver Operating Characteristic) curves for univariate logistic regression models calculated for patients who underwent END, with AUC representing the area under the curve. Optimal cut-off value determined by Youden index at 3 tumor buds

The probability of LNM in relation to changes in parameter units indicates that PTB (OR 1.43, CI 1.18–1.73) is a stronger predictor of LNM than tumor size (OR 1.008, CI 0.97–1.05; Fig. 4A). In patients who underwent END, those with LNM exhibited significantly higher PTB at the primary tumor site compared to patients who also received END but had no LNM (*p <* 0.01; Fig. 4B). For determining the indication for END, a subgroup analysis of patients with clinically negative lymph nodes (cN0) who underwent END was conducted. In this subgroup, significantly higher PTB was observed at the primary tumor site in pN + patients compared to pN − patients (Fig. 4C). Next, we applied a univariate logistic regression model to calculate the probability of LNM for all patients (Fig. 4D–E). PTB emerged as the strongest predictor of LNM, with an area under the curve (AUC) of 0.86, thereby outperforming previously established pathological scoring systems in our cohort. Using Youden index an optimal cut-off value of 3 tumor buds was determined.

### Pathological grading systems and prediction of LNM

A more detailed comparison of previously published pathological grading systems between pN + and pN − patients revealed significant differences between the two groups (Table 3, Supplementary Table 3). Agaimy's grading system showed no significant difference between both groups. In contrast, Bryne's grading, which assesses nucleus polymorphism (*p* = 0.017), invasion pattern (*p* = 0.016), and the resulting overall grade (*p* = 0.007), demonstrated significant differences. However, Boxberg's grading system appeared to be even more effective in predicting LNM, with significant differences observed for TB (TB; *p* = 0.0007), the smallest TB cluster size (*p* = 0.014), and the resulting grading (*p* = 0.005).

**Table 3:** Specific histopathological grading systems

	**Without neck dissection *n =* 24**	**pN0 *n =* 26**	**pN + *n =* 7**
Agaimy et al*.* (2016) [19]			
• Nucleus–To–Cytoplasm–Ratio			*p* = 0.33
◦ G1	6 (25.0%)	4 (15.4%)	0 (0.0%)
◦ G2	14 (58.3%)	18 (69.2%)	4 (57.1%)
◦ G3	4 (16.7%)	4 (15.4%)	3 (42.9%)
• Nuclear morphology			*p* = 0.05
◦ G1	8 (33.3%)	5 (19.2%)	0 (0.0%)
◦ G2	13 (54.2%)	17 (65.4%)	3 (42.9%)
◦ G3	3 (12.5%)	4 (15.4%)	4 (57.1%)
• Mitoses			*p* = 0.075
◦ G1	8 (33.3%)	7 (26.9%)	0 (0.0%)
◦ G2	13 (54.2%)	15 (57.7%)	3 (42.9%)
◦ G3	3 (12.5%)	4 (15.4%)	4 (57.1%)
• Stromal induction			*p* = 0.065
◦ G1	4 (16.7%)	2 (7.7%)	0 (0.0%)
◦ G2	18 (75.0%)	18 (69.2%)	3 (42.9%)
◦ G3	2 (8.3%)	6 (23.1%)	4 (57.1%)
• Keratinization			*p* = 0.79
◦ G1	5 (20.8%)	8 (30.8%)	1 (14.3%)
◦ G2	13 (54.2%)	13 (50.0%)	5 (71.4%)
◦ G3	6 (25.0%)	5 (19.2%)	1 (14.3%)
• Total Grading			*p* = 0.61
◦ G1	6 (25.0%)	5 (19.2%)	0 (0.0%)
◦ G2	15 (62.5%)	17 (65.4%)	5 (71.4%)
◦ G3	3 (12.5%)	4 (15.4%)	2 (28.6%)
Boxberg et al*.* (2017) [18]			
• Tumor buds (10 HPF)			***p* = 0.0007**
◦ 1 Point	14 (58.3%)	7 (26.9%)	0 (0.0%)
◦ 2 Points	6 (25.0%)	13 (50.0%)	1 (14.3%)
◦ 3 Points	4 (16.7%)	6 (23.1%)	6 (85.7%)
• Smallest tumor budding cluster size			***p* = 0.014**
◦ 1 Point	11 (45.9%)	5 (19.2%)	0 (0.0%)
◦ 2 Points	3 (12.5%)	2 (7.7%)	0 (0.0%)
◦ 3 Points	5 (20.8%)	11 (42.3%)	1 (14.3%)
◦ 4 Points	5 (20.8%)	8 (30.8%)	6 (85.7%)
• Total grading			***p* = 0.005**
◦ G1 (2–4 Points)	14 (58.3%)	7 (26.9%)	0 (0.0%)
◦ G2 (5–6 Points)	6 (25.0%)	13 (50.0%)	2 (28.6%)
◦ G3 (> 6 Points)	4 (16.7%)	6 (23.1%)	5 (71.4%)
Bryne et al*.* (1992) [20]			
• Keratinization			*p* = 0.67
◦ Score 1	2 (8.3%)	4 (15.4%)	0 (0.0%)
◦ Score 2	10 (41.7%)	9 (34.6%)	2 (28.6%)
◦ Score 3	6 (25.0%)	9 (34.6%)	2 (28.6%)
◦ Score 4	6 (25.0%)	4 (15.4%)	3 (42.8%)
• Nucleus polymorphism			***p* = 0.017**
◦ Score 1	7 (29.2%)	2 (7.7%)	0 (0.0%)
◦ Score 2	13 (54.2%)	19 (73.1%)	2 (28.6%)
◦ Score 3	3 (12.5%)	5 (19.2%)	4 (57.1%)
◦ Score 4	1 (4.1%)	0 (0.0%)	1 (14.3%)
• Invasion pattern			***p* = 0.016**
◦ Score 1	7 (29.2%)	3 (11.5%)	0 (0.0%)
◦ Score 2	4 (16.7%)	2 (7.7%)	0 (0.0%)
◦ Score 3	6 (42.3%)	11 (42.3%)	0 (0.0%)
◦ Score 4	7 (38.5%)	10 (38.5%)	7 (100.0%)
• Host response			*p* = 0.52
◦ Score 1	7 (29.2%)	10 (38.5%)	3 (42.9%)
◦ Score 2	16 (66.7%)	12 (46.1%)	3 (42.9%)
◦ Score 3	1 (4.1%)	4 (15.4%)	1 (14.2%)
• Total grading			***p* = 0.007**
◦ G1	13 (54.2%)	9 (34.6%)	0 (0.0%)
◦ G2	9 (37.5%)	16 (61.5%)	4 (57.1%)
◦ G3	2 (8.3%)	1 (3.9%)	3 (42.9%)

**Fig. 5:**
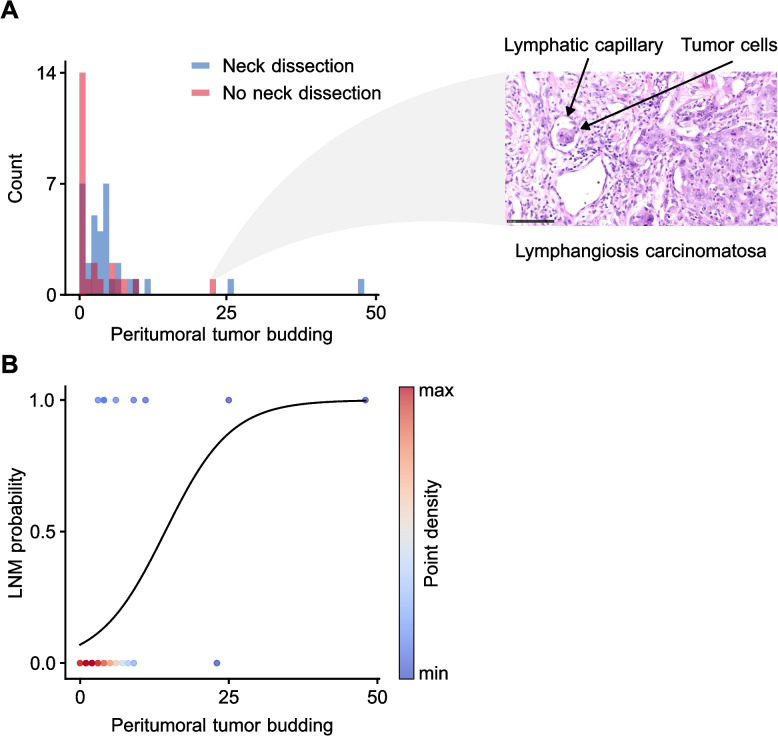
**a** Histogram depicting the number of patients with different numbers of peritumoral tumor buds. Outlier patients that did not receive END with high count of tumor buds displayed lymphatic infiltrations of tumor cells. A black scale bar of 100 µm is shown in the lower left corner of the image. **b** Probability of lymph node metastasis (LNM) according to peritumoral tumor budding with sigmoid regression line. Dots are colored according to density

Finally, the distribution of PTB between patients who received an END and those who did not was assessed (Fig. 5A). Patients who did not undergo END exhibited a lower maximum PTB compared to those who received an END. Interestingly, a single patient in the non-END group had an exceptionally high PTB count. Further investigation revealed that this patient also had lymphatic angioinvasion (L1) and could not undergo END due to comorbidities. The probability of LNM in follow-up increased markedly with higher levels of PTB (Fig. 5B).

## Discussion

### Strengths and weaknesses of the study design

The main limitations of this study include the small patient cohort and its retrospective design.

A key strength of this study is the high level of methodological standardization in the investigated parameters. This standardization should also include staining. TB analyses with immunohistochemical staining such as pancytokeratin have been described in the literature [22]. For example, differences between HE and pancytokeratin-immunostaining could be shown in laryngeal cancer outcome [23]. In this study, HE was mainly chosen because it is the standard stain in morphological pathology and is used worldwide. This would allow easy integration into a daily clinical workflow and could be used worldwide, even in countries with limited financial resources [16, 24]. A limitation of the present study is that TB assessment was performed by a single experienced pathologist without formal interobserver agreement analysis. However, the use of a standardized evaluation protocol and blinding to clinical outcomes minimized potential observer bias.

The HNSCC also showed divergent TB of different tumor infiltrations front sections. Cacchi et. al. showed that the TB scores of 50% of patients with HNSCC differed in different marginal sections in *n =* 66 patients with HNSCC. In addition, a TB average (TB rel) of the margin sections was analyzed to compensate for these differences [10]. This problem was additionally addressed in the LSCC by examining the highest TB of all the sections of tumor infiltration front examined in hot spot analysis. In addition, the indication of ITB and PTB also appears to be decisive. ITB could be identified as a tumor marker in colorectal carcinomas and therapeutic consequences could also be derived [16, 25]. Of note, that the increasing use and spread of digital technology in histopathology can facilitate the assessment of this (as well as) other histological parameters, with increasing precision and accuracy. Furthermore, it should be acknowledged that a selection bias may exist in patients who underwent neck dissection. Specifically, in the case with high TB but without neck dissection, the LNM rate may have been underestimated.

In this study, no comparison with the predictive efficacy of PD-1 or PD-L1 was performed. However, in the study by M. Klein et al., PD-L1 was shown to have no predictive value. Nevertheless, the majority of patients (> 50%) exhibited a PD-L1 expression of > 1%, indicating a potential response. It may therefore be possible that a combination of immune checkpoint analysis and TB analysis could also be applied therapeutically [8].

### Which is the best grading system for LSCC LNM prediction?

Grading according to Bryne and Boxberg appears to be a suitable analysis tool for LNM prediction in LSCC, as shown by the significant correlations. Other authors have also investigated grading and its influence on LNM in LSCC. For example, de P. Santos et al. compared three different grading systems for LSCC. On the one hand, the invasive front grading system (Grading Bryne et al.), the World Health Organisation (WHO) system and on the other, the histological risk assessment. No correlations were found for the WHO system. The invasive front grading system showed an association between low grade of malignancy and absence of LNM. In addition, a correlation between the risk score and LNM was found for the risk assessment. Immunological components were also investigated, so that lymphocytic infiltrates correlated with LNM. The authors postulate that histological risk assessment (Brandwein-Gensler et al.) is the best option for grading [12, 26]. The risk assessment was not examined in our study, but should also be included in a comparison in follow-up studies. In grading according to Boxberg, the TB is present to form the grading. The classical definition of TB has been established primarily in the context of colorectal carcinoma. In this entity, the TNM classification considers the anatomical structure involved at the site of deepest invasion as the relevant T parameter (analogous to Clark’s levels in malignant cutaneous melanoma). Notably, in early-stage disease (invasion confined to the submucosa (T1) or, at most, the muscularis propria (T2), without evidence of lymphatic or distant metastasis), the presence of TB is associated with an increased risk of lymph node metastasis. In carcinomas of the lip mucosa and skin, the depth of invasion becomes a relevant criterion for TNM classification only from a tumor thickness of 6 mm (T3) onwards. However, unlike in colorectal carcinoma, no distinct anatomical structures are available to further subclassify early stages of lip carcinoma. Consequently, detailed assessment of the maximum depth of invasion, with particular focus on TB in analogy to colorectal tumors, may provide valuable insights into the biological behavior of these neoplasms [27–30]. This is particularly relevant in T1 and T2 tumors, as this anatomical localization of carcinoma is considered to confer a higher risk of metastasis compared with other sites of cutaneous squamous cell carcinoma cSCC.

### TB is perhaps the starting point of local metastasis genesis

In one patient without neck dissection, massive lymphatic vessel invasion was observed in association with increased TB. As this observation is limited to a single case (*n =* 1), it should be interpreted cautiously and warrants further investigation in follow-up studies. Based on these findings, it can be hypothesized that TB may represent a mechanistic link between primary tumor localization, lymphatic vessel invasion, and the development of lymph node metastasis. An explanation for the exact mechanism, or whether this represented a coincidental observation, cannot be provided. Unfortunately, this case did not have sufficient follow-up to assess the occurrence of metastasis over time; therefore, this hypothesis should be further evaluated in future studies.

### TB should be included in every histopathological report for LSCC

TB should be simple to use for diagnostic and therapeutic purposes so that TB could be integrated into the pathological report in two different ways: either by stating the grading as TB in the Boxberg grading or as additional information in the histological report. In the authors' view, the grading should contain more additional information than TB. Therefore, grading according to Bryne is probably a more suitable tool, because of the specification of degree of keratinization, nuclear polymorphism, pattern of invasion and host response (infiltration of leukocytes). As a result, TB should be integrated into the tumor histological report as TB1/2/3, similar to Pn0/1, V0/1 and L0/1 status. In the German guideline for colorectal carcinoma, TB determination is recommended and should be included in the pathological report [31]. Xie et al. also recommended for HNSCC that TB should be included in every pathology report and that TB is a reproducible method [17]. For LSCC the evidence is not available, and most studies refer to HNSCC.

### Tumorbudding is a better predictor of LNM than previously established histomorphological parameters

The prediction of LNM in LSCC has been a subject of investigation for decades. In 1979, Shear et al. published one of the first comprehensive studies, noting that LNM is relatively rare in LSCC compared to other HNSCC [32]. Tumor size and differentiation were identified as the primary predictors of LNM in this study, a finding subsequently corroborated by numerous studies over the years [11, 33]. Other factors associated with LNM include depth of invasion and tumor diameter [11, 34]. After identifying TB as a LNM risk predictor, the indication for END should also be discussed, since LNM is correlated with significant reduced survival [4, 5]. This study evaluated more than 81 clinical and pathological parameters to identify more accurate predictors of LNM, thereby assisting surgeons in determining when to perform END. Among all analyzed parameters, PTB emerged as the most significant predictor of LNM. In this study, PTB outperformed these established grading systems as the strongest predictor of LNM in LSCC. Notably, the grading system proposed by Boxberg et al., which incorporates PTB, also highlights the importance of this feature. These findings suggest that LNM in LSCC is particularly driven by PTB. Additionally, numeric quantification of PTB demonstrated significant predictive advantages over categorical grading in the Boxberg system. At this point, it should be discussed that Boxberg summarises the number of buds in a score for practical reasons, thereby reducing the granularity of the data for the purpose of applicability. Boxberg grading is eliminated from the LASSO algorithm due to its high collinearity with the parametric tumor buds.

TB correlates with the risk of LNM in HNSCC and is also associated with a poorer outcome [35, 36]. Other studies also confirm that TB correlates with the risk of LNM in HNSCC and that therapeutic decisions could also be influenced [37, 38]. The indication for performing END in LSCC remains challenging to determine. LNM in LSCC occur less frequently than in other head and neck squamous cell carcinomas (HNSCC), complicating the selection of patients for elective nodal evaluation. Without END, the incidence of late LNM has been reported to be approximately 8% [39]. However, there is a need to minimize unnecessary END. In HNSCC a threshold value of 20% occult metastasis is required for the indication of an END to be beneficial [40]. Current cSCC guidelines support a conservative indication as the basis for decision-making in these cases. In contrast the oral cancer guideline supports an END in cN0 situation [2, 41].

While quantifying a total of 81 parameters per patient was labor-intensive, we feel that this laid a foundation for future studies, which should particularly focus on validating the prognostic value of PTB in larger, prospective cohorts. Furthermore, to enhance clinical utility, discussions regarding the inclusion of PTB in routine pathological reporting for LSCC should be based on data from larger samples.

By refining the prediction of lymph node metastasis, PTB offers a valuable tool for improving surgical decision-making in LSCC. Incorporating PTB into routine practice could help surgeons avoid unnecessary END in patients with a low likelihood of metastasis, while simultaneously improving outcomes for high-risk patients with increased TB. The data shown here emphasizes the benefit of LNM prediction in LSCC. On one hand, the analyses show that the presence of LNM increases TB values. In addition, the analysis of the cN0 neck showed an increased presence of TB. Therefore, it can be assumed that TB analysis is beneficial in preoperative cN0 necks and can assist in determining the need for END. This hypothesis should be validated in larger cohorts and prospective studies.

### Integration of TB into a clinical process between pathology and head- and neck surgery

An important factor for clinical implementation is also the feasibility in terms of time. This is influenced by the number of sections per case, additional parameters to be assessed in routine diagnostics, and the examiner’s level of experience. If no further parameters are evaluated, PTB can be assessed within approximately 1–2 min per section. However, a quantitative analysis was not performed in this study. The clinical integration of TB in colorectal carcinoma demonstrates that its implementation is also feasible [31]. The TB analysis should now be effectively integrated into a clinical workflow to determine the indication for END. Two different options are conceivable. The first option would be a preoperative biopsy with subsequent tumor resection planning with or without END in the cN0 neck. However, due to the tumor heterogeneity in the tumor infiltration front, this approach does not seem to make much sense in a single tumor infiltration zone [10]. In contrast, a two-stage approach could also be chosen. In the first step, the lip carcinoma is resected, and the subsequent TB analysis is performed from the highest hot-spot area of the tumor infiltration front from several sections. Then the highest TB subsequently determines the indication for elective neck dissection.

### High scientific potential for application in translational oncology

TB could perhaps be analyzed in LSCC in interdisciplinary translational oncology. For example, the response in dependence of TB during radiotherapy, systemic therapies or immunotherapies in advanced tumor stages could be investigated in future studies.

## Conclusion

PTB could help in the decision and indication process of elective neck dissection in LSCC. This hypothesis needs to be further validated in prospective study designs and larger patient cohorts.

## Supplementary Information

Supplementary Material 1: Supplementary Table 1. Grading-Systems. Supplementary Table 2. Software packages. Supplementary Table 3. Additional pathological patient data

## Data Availability

The data presented in this study are available on request from the corresponding author. There is a restriction under data protection regulation, so that the overall data is not publicly accessible.

## Abbreviations

ASA: American Association of Anesthesiologists
cSCC: Cutaneous Squamous Cell Carcinoma
DM: Distant Metastasis
END: Elective Neck Dissection
FFPE: Formalin-fixed paraffin-embedded
HNSCC: Head- and Neck Squamous Cell Carcinoma
HPF: High-power field
ITB: Intratumoral Tumor Budding
LASSO: Least Absolute Shrinkage and Selection Operator
LNM: Lymph Node Metastasis
LNR: Lymph Node Ratio
LSCC: Lip Squamous Cell Carcinoma
pN +: Pathological confirmed lymph node metastasis
pN–: Pathological negative for lymph node metastasis
PTB: Peritumoral Tumor Budding
TB: Tumor Budding
TIF: Tumor-invasion-front
TND: Therapeutic Neck Dissection
UICC: Union for International Cancer Control
