# Supplementary material for: Tumor budding predicts lymph node metastasis in squamous cell carcinoma of the lip
Source: Head Face Med. 2025 Nov 3;21:74. doi: 10.1186/s13005-025-00553-2 (PMC12581523; doi:10.1186/s13005-025-00553-2)
Supplement: Supplementary file 1 — Supplementary Material 1: Supplementary Table 1. Grading-Systems. Supplementary Table 2. Software packages. Supplementary Table 3. Additional pathological patient data [file 13005_2025_553_MOESM1_ESM.docx]

**Supplement**

**Supplementary Table 1.** Grading-Systems

| **Grading System** | **Criterion** |  |  |  |  |
| --- | --- | --- | --- | --- | --- |
| **Agaimy** |  | **G1** | **G2** | **G3** |  |
|  | **Nucleus-to-Plasma-Ratio** | Essentially preserved (comparable to normal squamous epithelium) | Moderatly shifted in favor of the nuclei | Strongly shifted in favor to the nuclei |  |
|  | **Nuclear morphology** | Slightly pleomorphic, open chromatin structure, single nucleoli | Moderatly pleomorphic, inhomogeneous chromatin structure | Strongly pleomorphic, severely disrupted chromatin structure |  |
|  | **Mitoses** | Few, peripheral in the cell nests, not atypical | Increased, scattered within the cell nests, few atypical mitoses | Numerous, dispersed, often atypical |  |
|  | **Stromal induction** | Minimal | Moderate | Strong |  |
|  | **Keratinization** | Strong, differentiated/ directed | Weak to moderate, aberrant | Almost absent |  |
|  | **Additional aspects** | Cytomorphologically almost like „normal“ squamous epithelium | / | Morphologically identifiable only in parts as a squamous cell carcinoma |  |
| **Boxberg** |  | **1 Point** | **2 Points** | **3 Points** | **4 Points** |
|  | **Tumor buds (10 HPF)** | None | < 15 tumor buds | > 15 tumor buds | / |
|  | **Smallest tumor cell nest size** | > 15 cells | 5 – 15 cells | 2 – 4 cells | Single cell infiltration |
| **Bryne** |  | **Score 1** | **Score 2** | **Score 3** | **Score 4** |
|  | **Degree of keratinization** | Highly keratinized (> 50 % of the cells) | Moderatly keratinized (20-50 % of the cells) | Minimal keratinization (5-20 % of the cells) | No keratinization (0-5% of the cells) |
|  | **Nuclear polymorphism** | Little nuclear polymorphism (> 75 % mature cells) | Moderatly abundant nuclear polymorphism (50-75% mature cells) | Abundant nuclear polymorphism (25-50 % mature cells) | Extreme nuclear polymorphism (0-25 % mature cells) |
|  | **Pattern of invasion** | Pushing, well delineated infiltrating borders | Infiltrating, solid cords, bands and/or strands | Small groups or cords of infiltrating cells (n>15) | Marked and widespread in small groups and/or single cells (n<15) |
|  | **Host response (infiltration of leukocytes)** | Marked | Moderate | Slight | None |

**Supplementary Table 2.** Software packages

| **Package** | **Version** | **References** |
| --- | --- | --- |
| Matplotlib | 3.8.0 | (46) |
| NumPy | 1.26.4 | (47) |
| pandas | 2.1.4 | (48) |
| seaborn | 0.12.2 | (49) |
| scikit-learn | 1.2.2 | (50) |
| SciPy | 1.11.4 | (51) |
| statsmodels | 0.14.0 | (52) |

**Supplementary Table 3.** Additional pathological patient data.

|  | **END cohort**  **n = 33** | **pN0**  **n = 26** | **pN+**  **n = 7** |
| --- | --- | --- | --- |
| Ulceration^†^   - Yes - No | 28  5 | 21  5 | p = 0.51  7  0 |
| Necrosis^†^   - Yes - No | 6  27 | 3  23 | p = 0.18  3  4 |
| Muscle infiltration^†^   - Yes - No | 23  10 | 16  10 | p = 0.13  7  0 |
| Salivary gland infiltration^†^   - Yes - No | 5  28 | 2  24 | p = 0.08  3  4 |
| Bone infiltration^†^   - Yes - No | 0  33 | 0  26 | p = 1.0  0  7 |
| Tumor-Stroma-Ratio (%)* | 0.70 ± 0.20 | 0.70 ± 0.20 | **p = 0.029**  0.50 ± 0.15 |
| Worst pattern of invasion^†^   - Yes - No | 1  32 | 1  25 | p = 1.0  0  7 |
| Mitosis (Count / 10 HPF)* | 16.5 ± 16.25 | 15.0 ± 13.0 | p = 1.0  23.0 ± 14.5 |
| Eosinophils (Count / 10 HPF)* | 35.0 ± 57.0 | 48.50 ± 66.25 | p = 0.55  35.00 ± 23.50 |
| Solar elastosis^†^   - Yes - No | 33  0 | 26  0 | p = 1.0  7  0 |
| * Median ± interquartile range (IQR) † Count data | | | |
